# Kinetics and adsorption isotherm of cadmium and lead on waste- agricultural derived biochar and their stabilization in two different polluted soils

**DOI:** 10.1038/s41598-026-67905-z

**Published:** 2026-09-05

**Authors:** Laila R. Salem

**Affiliations:** Water Resources and Desert Soils Division, Department of Soil Chemistry and Physics, Desert Research Center, El-Matareya, Cairo, Egypt

**Keywords:** Sugarcane bagasse biochar, Heavy metals, Adsorption, Plant uptake, Environmental sciences, Plant sciences

## Abstract

The current study evaluated sugarcane bagasse biochar (SCBB) as an adsorbent for cadmium (Cd²⁺ ) and lead ( Pb²⁺). By using the stirred-batch technique, a kinetic experiment was conducted at two concentrations, while an adsorption isotherm experiment was performed at five concentrations. Furthermore, the role of SCBB at doses (0%, 0.5%, 1%, 2%) for stabilization of Cd^2+^ and Pb^2+^ in two polluted soils (S(I), S(II)) and decreasing their bioavailability was assessed through pot experiment. Results showed that equilibrium was achieved after 2 h for Cd^2+^ and after 1 h for Pb^2+^. Pseudo second-order model was more fitted for kinetic data (R²= 0.99) for Cd²⁺ and Pb²⁺ at two concentrations; thus, chemisorption may controladsorption process. Adsorption isotherm results were better represented by the Langmuir model (R²= 0.99). Consequently, monolayer adsorption may be the main mechanism in Cd²⁺ and Pb²⁺ adsorption. The efficiency of Pb²⁺ removal by SCBB was higher than that of Cd²⁺, where it was 60%, while for Cd²⁺ it was 50% at the same initial concentration. Cultivation experiment demonstrated that SCBB could significantly decrease Cd²⁺ and Pb²⁺ uptake by radish plants. The percentage of decrease in Cd²⁺ content in shoot was 49% and 54%, while it was 57% and 45% for Pb^2+^ at SCBB 2% in S(I) and S(II), respectively. A positive correlation between DTPA- extracted Cd^2+^ and Pb^2+^ in soil and their content in plant was obtained. An increase in CEC, OM of the agricultural environment, and dry biomass of shoot and root was observed. Therefore, resourceful utilization of SCBB is a beneficial method for removing lead and cadmium from aqueous solutions and stabilizing them in polluted soil.

## Introduction

Urbanization and industrialization increase the release of organic and inorganic waste products into the environment, which is considered a global problem^1^. About 0.9 and 1.4 billion people live in heavy metal-polluted worldwide agroecosystem zones, which pose considerable ecological and health risks^2^. Heavy metals contamination has increased as a result of human activities, that have degraded water quality, endangering ecosystems, and public health. These activities include mining, cultivation, urbanization, industrialization, and unsustainable resource use^3–6^. Heavy metals such as Pb²⁺, Cd²⁺, Cu²⁺, Fe, and Zn²⁺ enter water environment through sewage and industrial waste discharge with long-term health and environmental consequences^7,8^. In addition, heavy metals have high persistence in soil, consequently they enter food chains via water and crops, endangering plant and human health^9–11^. More than 90% of cancers are associated with chemical exposure, including aquatic pollutants^12^ because these metals elicit carcinogenic, genotoxic, and cytotoxic effects^13^. Certain metals have particular hazards for instance, lead increases blood pressure^14^ and acts as neurotoxins^15^. Lead and cadmium are two of the top ten poisonous compounds listed by the World Health Organization, and their devastating effects on humans and other living things are well documented. Cadmium may seriously damage the kidneys, and lead is a very toxic metal, that can impact nearly every organ and system in the body, particularly the nervous system^16^. For plants As²⁺, Cd²⁺, Hg²⁺, and Pb²⁺ are non-essential metals and inhibit photosynthesis, destroy DNA, and render enzymes inactive^17–20^. Through several mechanisms, heavy metals in soil can have detrimental effects on groundwater, microbial diversity, soil quality, and agricultural production^21,22^. Municipal wastewater irrigation increases soil and crop metal levels, increasing contamination and endangering consumers^23,24^. In Egypt agriculture depends mostly on fresh water, which is increasingly scarce due to population growth, diminishing capacity of surface water reservoir, and an unmanaged irrigation water supply system. Therefore, Egypt makes use of polluted water from the drain in agricultural soil. It is necessary to employ drainage effluent, such as from the Bahr El Baqar drain, for the cultivation of agricultural crops, particularly in urban agricultural lands where, water is scarce. The Bahr El-Baqar drain receives waste water from its starting point east of Cairo, at El-Gebel El-Asfar, and then is connected by the Belbeis drain down to the Qalubiya drain. The calculated water quality index (WQI) classified the water in the Bahr El-Baqr drain as poor drainage water^25^. Given that the Bahr El Baqar drain is regarded as one of Egypt’s most contaminated drains, reusing this drainage water could negatively impact soil, crops, animals, and human health^26,27^. Using this contaminated water for irrigation introduces poisons into soils and crops^28^. The Bahr El Baqar drain’s outflow of municipal, industrial, and agricultural wastewater contaminated the surrounding area with a variety of contaminants, including heavy metals such as lead and cadmium^29^. According to the findings of the soil analysis, mentioned by Elbana et al.^30^ Pb²⁺ concentrations in the soils were marginally higher than usual, while Cd²⁺ contamination was noted. On the other hand, in the Burg Al Arab area, some agricultural soil has been irrigated with waste industrial water. Detergent manufacturing, food processing, and textiles are the main industrial activities there. Thus, there has been heavy metal pollution in these soils^31^.

Nowadays, the main methodologies for remediating soils contaminated with Cd²⁺ and Pb²⁺ include chemical, physical, biological, and integrated remediation strategies^32^. Various types of amendments have been developed and applied for remediation of heavy metals in contaminated soil, such as iron and ferric salts^33^, bentonite, zeolite^34^, red mud^35,36^, silicon, and modified silicon^37,38^. These amendments reduce the availability of heavy metals to plants due to their activity decline^36,37^. However, these amendments have several issues including poor specificity, inefficiency, potential contamination from secondary sources, and economic hurdles^33,39^. Subsequently, to treat heavy metal-contaminated soils, new amendments such as biochar (BC) have been proposed as an effective, specific, and less negatively impacting on the soil environment. Biochar and charcoal are both carbon-rich materials produced through Pyrolysis. However, biochar is generally produced under controlled conditions for environmental applications such as soil improvement and pollutant adsorption, whereas charcoal is traditionally manufactured as a fuel material. Nevertheless, the two materials can have similar physicochemical properties such as high carbon content, porous structure, and surface functional groups which contribute to their adsorption behavior as mentioned by Narkpiban et al.^40^ in a study of Bamboo charcoal production. The use of biochar in soil remediation has grown significantly; it is a waste- biomaterial formed from the thermal decomposition of biomass in an oxygen-deficient environment^41–44^. It is a porous carbon - rich biomaterial; recently it has been applied to contaminated soil to stabilize heavy metals in soil and decrease their plant uptake^45–48^. Puga et al.^49^ stated that applying biochar made from sugarcane straw reduces the availability of Cd²⁺, Pb²⁺, and Zn²⁺ in soil by 56.5%, 50.0% and 54.0%, respectively. Biochar reduced the bioavailable Pb²⁺, Cd²⁺, and Zn²⁺ by 92%,71%, and 87%, respectively^50^. In addition to stabilizing carbon and enhancing soil qualities, applying biochar to polluted soil can reduce the toxicity of metals and address heavy metal contamination^42,51–53^. Because of its recalcitrant carbon, biochar also enhances soil characteristics and has a lengthy half-life^54,55^. Additionally, good porosity and fundamental functional groups in biochar make it an effective sorbent for gas pollutants in flue gas^56^. Sugarcane bagasse (SCB) is a fibrous lignocellulose residue of the sugar production process^57,58^. It is inexpensive, carbon - rich and available in abundant quantities, making it suitable for the production of biochar^59^. About 4.7 million tons of sugarcane bagasse are produced each year; this large amount of waste creates challenges for the environment^60^. On the other hand, it is considered a useful tool for encouraging environmental sustainability. Salem et al.^61^ and Salem^62^ demonstrated that SCBB is a.

good amendment to stabilize Cu²⁺, Zn²⁺, Cr²⁺, and Ni²⁺ in contaminated soil, in addition to Sr²⁺ in polluted water and soil, and accordingly reduce their bioavailability. Otherwise, there is still a lack of research on the systematic measurement of how the dose of sugarcane bagasse biochar (SCBB) regulates the speciation Pb²⁺ and Cd²⁺, as well as its relationship to changes in soil physicochemical properties (i.e., CEC, organic carbon, and pH), while the plant content of these heavy metals remains under explored. Furthermore, even when the biochar was made from the same feedstock, its stabilization efficiency varied significantly. Both Bian et al.^63^ and Jia et al.^64^ employed biochar from wheat straw (550 °C) to immobilize Cd²⁺ in soils, and they found that the efficiency of stabilizing ranged from 18.8% to 54.5%. This is mostly explained by the different properties of the soil and the biochar^65^. Additionally, biochar addition does not always provide a favorable outcome^21,66^, and studies have reported adverse impacts of biochar on heavy metal-contaminated soil, demonstrating either increase metal solubility or no stabilizing effects in specific scenarios^67–70^.

Thus, the goals of this research were to examine the positive effect of sugarcane bagasse biochar as an adsorbent material to cadmium and lead in aqueous solutions and decline their uptake by plants through stabilizing them in polluted soil. Furthermore, dispose of sugarcane bagasse rubbish in an appropriate approach. These goals have been accomplished by studying the effect of biochar made from sugarcane bagasse on the adsorption of cadmium and lead from aqueous solutions to investigate reaction mechanisms and understand the rate at which reactions achieve equilibrium, and understanding how sugarcane bagasse biochar stabilizes the two elements and reduces their plant uptake in two different polluted soils through pots experiment.

## Materials and methods

### Preparation and characterization of waste- biomaterial biochar

Sugarcane bagasse (SCB) is a widely accessible agri-food waste in Egypt. The feedstock was washed with tap water, then with distilled water, to get rid of dirt and sand particles. SCB was air-dried and then oven-dried at 70 °C for 24 h. The pyrolysis process was performed by using a traditional method^62^. The feedstock was placed in a stainless-steel mesh, which was then loaded into a tightly sealed barrel within the pyrolysis unit, where it was heated under limited oxygen conditions for 2 h. The temperature was maintained at approximately 500 °C. After the pyrolysis process, sugarcane bagasse biochar (SCBB) was obtained. Once naturally cooled, the biochar sample was cleaned with distilled water to get rid of any contaminants and dried for five hours at 105 °C. After cooling the sample of biochar was broken down and sieved through a 0.5-mm sieve made of polypropylene.

FTIR (Fourier Transform Infrared) analysis was performed to identify the surface functional groups. The SCBB sample was scanned with infrared radiation in the range of 400–4000 cm⁻¹ by using a SHIMADZU FT/IR-5300 infrared spectrophotometer (JASCO Corporation, Japan). Scanning Electron Microscopy (SEM) was performed on the biochar sample to examine its surface morphology. The analysis was conducted using a SEM Quanta FEG unit with an accelerating voltage of 30 kv, (magnification 250 × up to 20,000 0061nd resolution for Gun.1 m).

Several physicochemical characteristics of SCBB were determined. Combustion oven-dried biochar at 500 °C for 12 h was done to determine ash content. 10 mL of HCl and HNO_3_ (3:1) acid mixture were used as a digestive solution to ash according to Jones and Case^71^ to extract heavy metals and then measured by Inductively coupled argon plasma optical emission spectrometry (ICAP 6500 Duo, Thermo Scientifc, England). By using a CHNS elemental analyzer (Vario EL, Elementar) the mass percentages of carbon (C), hydrogen (H), nitrogen (N), and sulfur (S) were measured. Percentage of O = 100 - (Ash%+H% + C%+ N%)^72^. The H/C, O/C, and (O + N)/C atomic ratios were determined in order to assess the aromaticity and polarity of SCBB. At a 1:20 w/v biochar-to-water ratio, the biochar’s electrical conductivity (EC) and pH were measured. According to the method of Song and Guo^73^, cation exchange capacity (CEC) was determined.

### Chemicals

Cadmium as Cd(NO_3_)_2_·4H_2_O and Lead as Pb(NO_3_)_2_ were purchased from Sigma Aldrich Company (Germany). Hydrochloric acid, nitric acid, Sodium hydroxide, Ammonium acetate, sodium acetate, Hydroxide amine hydrochloride, sodium chloride, potassium hydroxide, ferric sulfate, ethanol and calcium chloride, were obtained from Egypt, El-Gomhoria chemicals Co.

### Adsorption of cadmium and lead onto SCBB

The stirred-batch approach with water path circulation jacket at a constant-temperature of the 25 °C and 400 rpm was used for adsorption experiments. Experiments were conducted in triplicate, and the average values were reported, and for quality control, a blank solution was measured. Aqueous solutions of Cd(NO₃)₂·4 H₂O and Pb(NO₃)₂ were mixed separately as a test for mono-metal sorption with SCBB in a 1:200 w/v ratio (5 g/L) with 0.01 M NaNO₃ as a background electrolyte.

### Adsorption kinetics experiments

Kinetic experiments of Cd²⁺ and Pb²⁺ ions onto SCBB were carried out at two concentrations (low and high )for each ion to study the impact of initial metal concentration on adsorption behavior (around 5 and 25 mg/L Cd²⁺) and (around 25 and 50 mg/L Pb²⁺) at different residence times. By a polyethylene syringe, suspension was withdrawn from the reaction vessels after various desired contact times (10, 15, 30, 60, 120, 240, 360, 480, 720, 1200, and 1440 min).

### Adsorption isotherm

Adsorption isotherm studies of Cd^2+^ and Pb^2+^ were carried out at 25 °C in five gradient concentrations around (5–10 -20–40 -50 mg. L^−1^) for Cd^2+^ and Pb^2+^ at the equilibration time derived from previous kinetic studies for each element.

After kinetic and adsorption isotherm experiments, each mixture was centrifuged for five minutes before being filtered through a Whatman filter paper No. 42 to split up SCBB from aqueous solutions. In the supernatants, pH was measured instantly for treated and untreated Cd^2+^, Pb^2+^ solutions. Cd^2+^, Pb^2+^ were quantified by using ICAP 6500 Duo (Thermo Scientifc, England) .

### Data analysis

**The adsorptive quantity** q_t_ (mg. g^−1^) of Cd^2+^ or Pb^2+^ was calculated by the following equation for a particular quantity of adsorbent at time t.

$$\left( {{q_t}} \right)=V\;\left( {{C_i}-{\text{ }}{C_f}} \right)/M$$

(C_i_), (C_f_) are initial and final concentrations of the Cd^2+^ or pb^2+^ in solution (mg. L^−1^). V (L) and M (g) are the volume of sample and mass of SCBB, respectively.

**Percentage of removal **was calculated by using the following equation:

$$\% \;{\mathrm{of}}\;{\mathrm{Removal}}=\left( {\left( {{C_i}-{\text{ }}{C_f}} \right)/{C_i}} \right) \times 100$$

where C_i_ and C_f_ (mg. L^−1^) are the concentrations of Cd^2+^ or Pb^2+^ before and after adsorption time, respectively^74^.

Three mathematical models were applied for characterizing kinetic of Cd^2+^ and Pb^2+^ sorption by SCB biochar (pseudo-first order, pseudo-second order and Elovich models in linear form)^75^.

**Pseudo-first-order model**

$$Ln{\text{ }}\left( {{q_e} - {q_t}} \right)=\ln {\text{ }}{q_e} - {k_1} \cdot t$$

where q_e_ and q_t_ (mg. g^−1^) are quantities of Cd^2+^ or Pb^2+^ adsorbed onto the biosorbent material (SCBB) at equilibrium and at the specified time (t (h)), respectively. k_1_ (h^−1^) is the adsorption rate constant of the pseudo first-order equation. The quantity of adsorbed ions and rate constant were calculated from the intercept and slope of the linear plot of t versus ln (q_e_ − q_t_).

**Pseudo-second order model**

$$t/{q_t}=1/\left( {{k_2}q_{e}^{2}} \right)+t/{q_e}$$

where q_e_ and q_t_ (mg. g^−1^) are mounts of Cd^2+^ or Pb^2+^ adsorbed onto bio sorbent material (SCBB) at equilibrium and at the selected time (t (h)), respectively. k_2_ (g.mg^−1^.h^−1^) is the rate constant of pseudo-second order equation.

**Elovich equation**

$${q_t}={\text{ }}(1/\beta ){\text{ }}\ln {\text{ }}(\alpha /\beta )\,+\,1/\beta \ln {\text{ }}T$$

where q_t_ (mg. g^−1^) is the quantity of adsorbed Cd^2+^ or Pb^2+^ onto SCBB at the selected resident time β (mg.g^−1^.min^−1^) and α (g.mg^−1^.min^−1^) are constants that will be calculated from the linear plot of ln T versus q_t_, where the slope (1/β) and the intercept (1/β) ln (α/β).

**Langmuir and Frendlich models**

Langmuir and Freundlich models were employed to explain the equilibrium data of Cd^2+^ and Pb^2+^ on SCBB^74,76^.

Langmuir equation (linear form)

$${C_e}/{q_e}={\text{ }}1/\left( {{q_{\hbox{max} }}{K_L}} \right)+{C_e}/{q_{\hbox{max} }},$$

where C_e_ (mg. L^−1^) is the concentration of cadmium or lead ions at equilibrium, q_e_ (mg. g^−1^) is the quantity of adsorbed Cd^2+^ or pb^2+^ onto SCBB, at equilibrium, q_max_ (mg. g^−1^) is the maximum adsorption capacity of Cd^2+^ or pb^2+^ onto SCBB and K_L_ (L.mg^−1^) is the Langmuir energy-related constant of adsorption.

The favorability of the ions’ adsorption onto the adsorbent materials could be assessed using Langmuir constant K_L_(L.mg^−1^)^77^. by using the following equation:

$${R_L}=1/\left( {1+{K_L}} \right) * {C_0},$$

Where R _L_ is the dimensionless constant separation factor, K_L_ (L.mg^−1^) is the Langmuir constant and C_0_ (mg. L^−1^) is the initial concentration of ions.

R _L_= 0 suggests that the adsorption process is irreversible.

R _L_ = 1 suggests that the adsorption is linear.

R _L_ > 1 suggests that the adsorption is unfavorable.

1 > R_L_> 0 suggests that the adsorption is favorable.

Freundlich isotherm model

$$Log\;{q_e}=1/n\;\log {\text{ }}{C_e}+\log {\text{ }}{K_F},$$

where C_e_ (mg. L^−1^) is the concentration of the cadmium or lead ions at equilibrium, q_e_ (mg. g^−1^) is the quantity of adsorbed ions onto SCBB at equilibrium, K_F_ is the adsorption constant of the Freundlich model, and n (dimensionless) is the empirical constant describing the nonlinearity of adsorption.

### Soil sampling and soil properties

Soil samples were collected at 0–30 cm depth from the Sahl El Husseiniya region S(I) and Burg Al Arab area S(II). The soil samples in S(I) were collected within 30° 50′ 48″ N latitude and 31° 58′ 53″ E longitude. This soil is irrigated mainly from the Bahr El-Baqar drain, which has wastewater. In the Burg Al Arab area S(II) samples were collected within 30° 53′ 13″ N latitude and 29°36′ 36″ E longitude. S(I) and S(II) have been polluted with heavy metals like cadmium and lead^31^. According to Black^78^, many chemical and physical analyses of soil were done. In soil: water suspension 1:2.5 (w/v), electrical conductivity (EC) was measured to determine soil salinity (dS/m) by using the Jenway conductivity meter model 4310 Jenway; pH of soil was determined by the pH-meter model 3305. The Scheibler method, Royal Eijkelkamp calcimeter, Giesbeek, Netherlands, was used to detect CaCO_3_ content in soil. Robinson’s pipette method (Eijkelkamp Agriresearch Equipment, Giesbeek, Netherlands) was used to analyze soil particles to the fraction less than 2 mm. Wet oxidative method^79^ was used to measure soil organic matter (SOM). By using the ammonium acetate method, cation exchange capacity (CEC) of soil was measured. Additionally, available concentrations of Cd, Pb, Fe, Ni, Cu, Mn, and Co were extracted using the DTPA method according to Lindsay and Norvell^80^, while total concentrations were extracted as described by Ure^81^. Then the elements were determined by ICAP 6500 Duo (Thermo Scientific, England).

### Planting experiment

In open field conditions, a pot experiment was carried out to study the efficiency of biochar addition on the bioavailability of cadmium and lead ions in the two soils, S(I) and S(II). The design of the experiment was a completely block randomized design; the experiment was conducted using soil samples from the Sahl El Husseiniya region (S(I)) and the Burg Al Arab Second industrial area (S (II)). In these experiments, radish plants (*Raphanus* *Sativus* *L*.) were used as test plants for cadmium and lead bioavailability during the remediation of polluted soil^82,83^. In plastic pots, three kilograms of air- dried soil were mixed with the SCBB at four different doses: 0%, 0.5%, 1%, and 2% of SCBB. Six seeds were sown in each pot and water was added to increase the soil moisture content to 70% of its water-holding capacity. Each treatment had three replicates. Fifty days after seeding, the plants were harvested.

### Soil and plant analysis

After plant maturity, soil was collected from each pot and air-dried, then prepared to proceed with some physicochemical properties according to Black^78^, like pH, EC and CEC, as well as OM that was measured according to the Walkley and Black^79^ method. In addition to available content of Cd²⁺ and Pb²⁺that was extracted by DTPA method according to Lindsay and Norvell^80^. As well, total content of Cd²⁺ and Pb²⁺ was extracted as described by Ure^81^. These elements were measured by inductively coupled argon plasma optical emission spectrometry ICAP 6500 Duo (Thermo Scientifc, England).

The Radish plant (*Raphanus Sativus*) was selected as a bio indicator. The whole plant was washed consecutively with tap and distilled water to get off dust or soil particles. The test plant was divided in two portions: above-ground portion (shoot) and underground portion (root). After both parts of the plant were air- dried, they were dried at 70 °C for 48 h in an oven. For each part of the plant, the dry weight was recorded for each pot. By using a stainless-steel grinder, both parts were ground. The digestion process was performed according to Jones^84^. Cd²⁺, Pb²⁺, Cu²⁺, Mn²⁺, Ca²⁺ and Mg²⁺ content was determined using ICAP 6500 Duo (Thermo Scientific, England).

### Statistical analysis

The Shapiro-Wilk test was used to confirm the normality of the data (*p* > 0.05)^85^. A two-factor analysis within a RCBD was performed using the GLM procedure in GenSTAT v.11 (VSN International Ltd., Oxford, UK). Treatment means were separated using Duncan’s multiple range test at *p* ≤ 0.05^86^. Pearson correlation matrices were generated and visualized using R software v.4.0.2.

## Results and discussion

### Characterization of waste – agricultural biochar

**Fig. 1:**
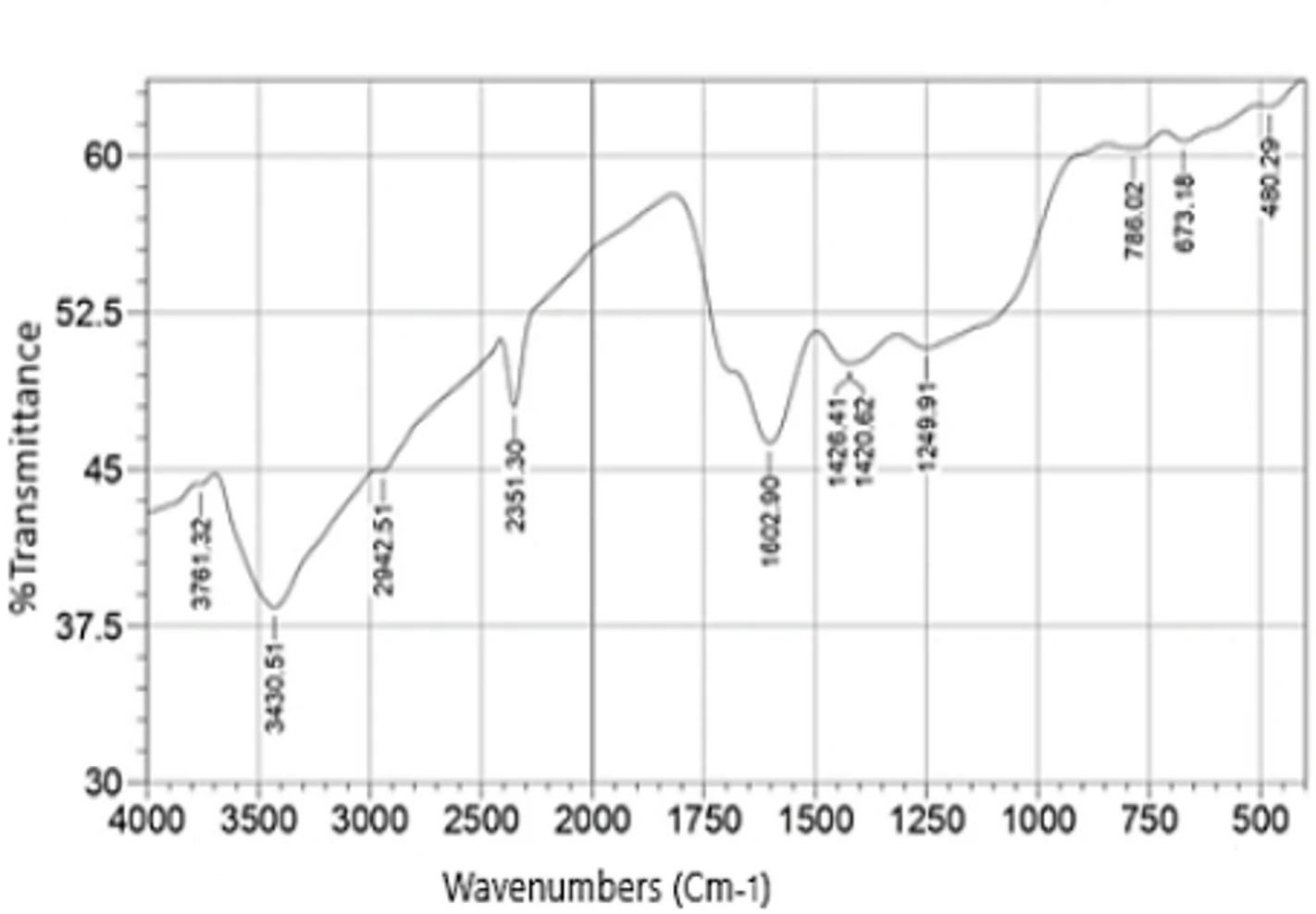
Fourier-transformed infrared spectrum (FTIR) of the SCBB^62^.

**Fig. 2:**
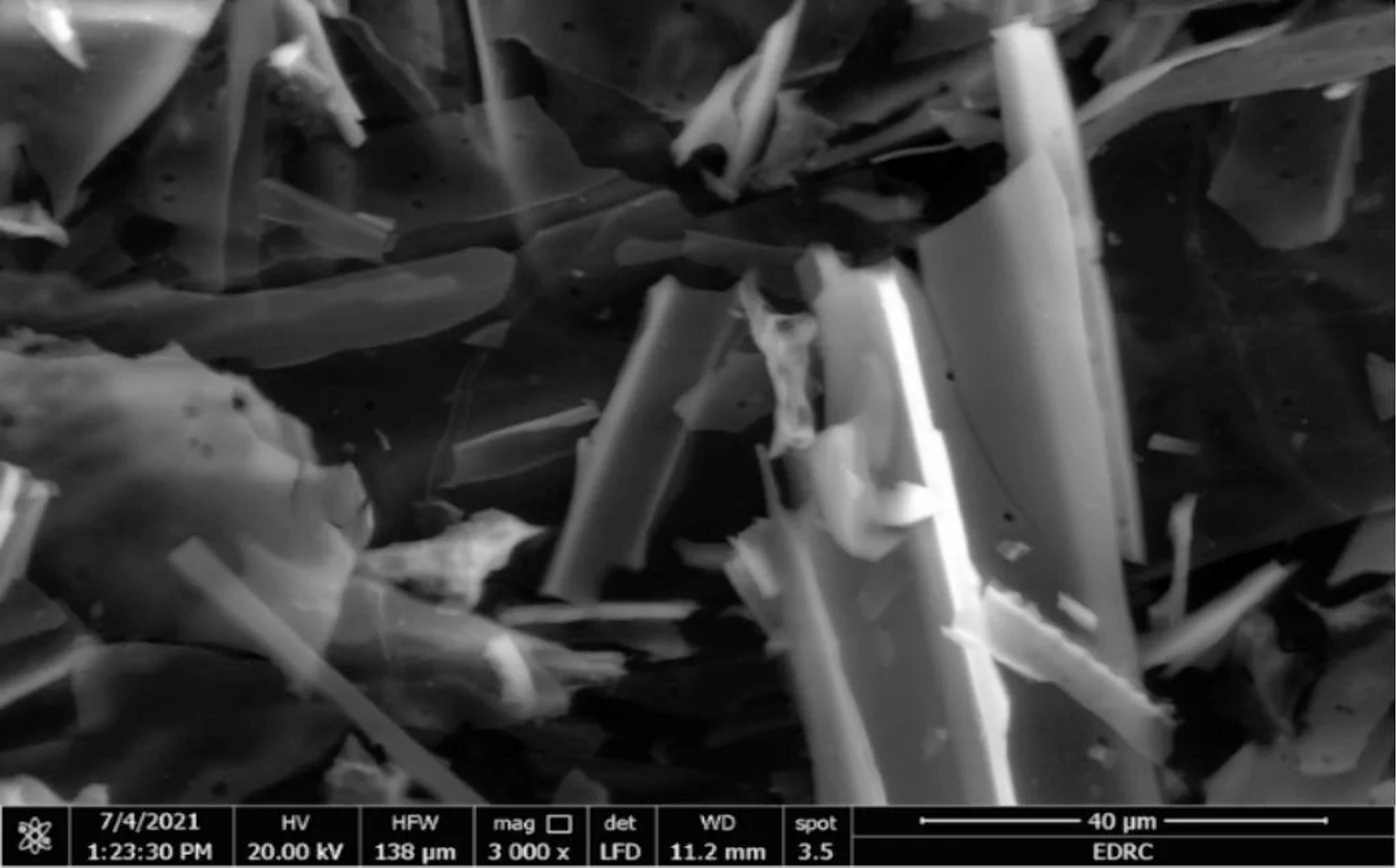
Scan electron microscope image (SEM) of SCB biochar^62^.

SCBB used in this study was characterized in my previous study Salem^62^. Some physicochemical characterization of SCBB are mentioned in Table 1 to elucidate its characteristics. The data showed that carbon, followed by oxygen, are the most common elements, as assured by El-Damarawy et al.^87^. SCBB has high ratios of O/C (0.344) and (O + N)/C (0.351), indicating that, SCBB has high polarity^88^. Additionally, SCBB has a high specific surface area (167.30 m^2^/g) and porous structure based on total pore volume (0.13 cm^3^/g), and average diameter of the pores (2.83 nm). Plant cells form tubular structures, which create this particular type of porosity^49^. SCBB has a significant degree of carbonization and strong aromaticity based on atomic ratios of H/C, O/C, and (O + N)/C, which indicates excellent metabolic stability, where the H/C ratio is less than 0.6^89^. Functional group compositions of SCBB were clarified by Fourier transform infrared spectra (FTIR) Fig. 1. For the SCBB, ten radiation spectra in the 4000–400 cm^−1^ range were observed. Peaks that were observed at 3430.51 cm^−1^ and 1602.90 cm^−1^ may be assigned to O–H stretching alcohol and C = C, C = O stretching carbonyl, respectively, as mentioned by Shin et al.^90,91^; Feng et al.^92^; Sahu et al.^93^. The spectrum at frequency 2351.30 cm^−1^ can be indicated to C ≡ C triple bonds^94^. In the nearby area of 786.02 and 673.18 cm^−1^ of C–H alkyl bound and aromatic compounds were represented. Furthermore, it can be explained as Si–O–Si and Si–OH (siloxane and silanol) reactive groups^95^. These results agreed with the composition of the elements’ findings. The functional groups, such as carbonyl and C ≡ C triple bond, can interoperate adsorption of Cd⁺² and Pb⁺² by SCBB. The surface morphology of SCBB had been shown by the scanning electron microscope image Fig. 2. The image showed the smooth surface of SCBB. In addition, SCBB has a large number of irregularly sized pores and canals which, means that it consists of meso- and macroporous structures as a result of the thermal degradation the volatile compound of SCB during the pyrolysis process^60,96^. This structure plays a vital role in the adsorption process because it increases their specific surface area.

**Table 1:** Some physical and chemical characteristics of sugarcane bagasse biochar (SCBB)^62^.

Properties	Value
Ash %	19.89 ± 0.84
pH	6.61 ± 0.06
EC dS m^−1^	0.43 ± 0.02
CEC meq/100 g	48.3 ± 0.17
SSA m^2^/g	167.30 ± 90
Carbon (C)%	54.64 ± 0.12
Hydrogen (H) %	6.13 ± 0.01
Oxygen (O) %	18.80 ± 0.15
Nitrogen (N)%	0.36 ± 0.02
Sulfur (S)%	0.21 ± 0.09
H: C	0.12 ± 0.03
O: C	0.344 ± 0.13
(N + O)/C	0.35 ± 0.001
Total pore volume cm^3^/g	0.13 ± 0.001
Average pore diameter nm	2.83 ± 0.01

CEC cation exchange capacity, SSA specific surface area, EC electrical conductivity, pH and EC measured in 1:20 w: v biochar water suspension (average ± STDEV, *n* = 3).

### Kinetics study of cadmium and lead on SCBB

A kinetic study reveals the effect of contact time on the adsorption process, interprets the mechanism of the adsorption process and how quickly reactions approach equilibrium. During these experiments pH was ranged between 6.35 and 6.97.

**Fig. 3:**
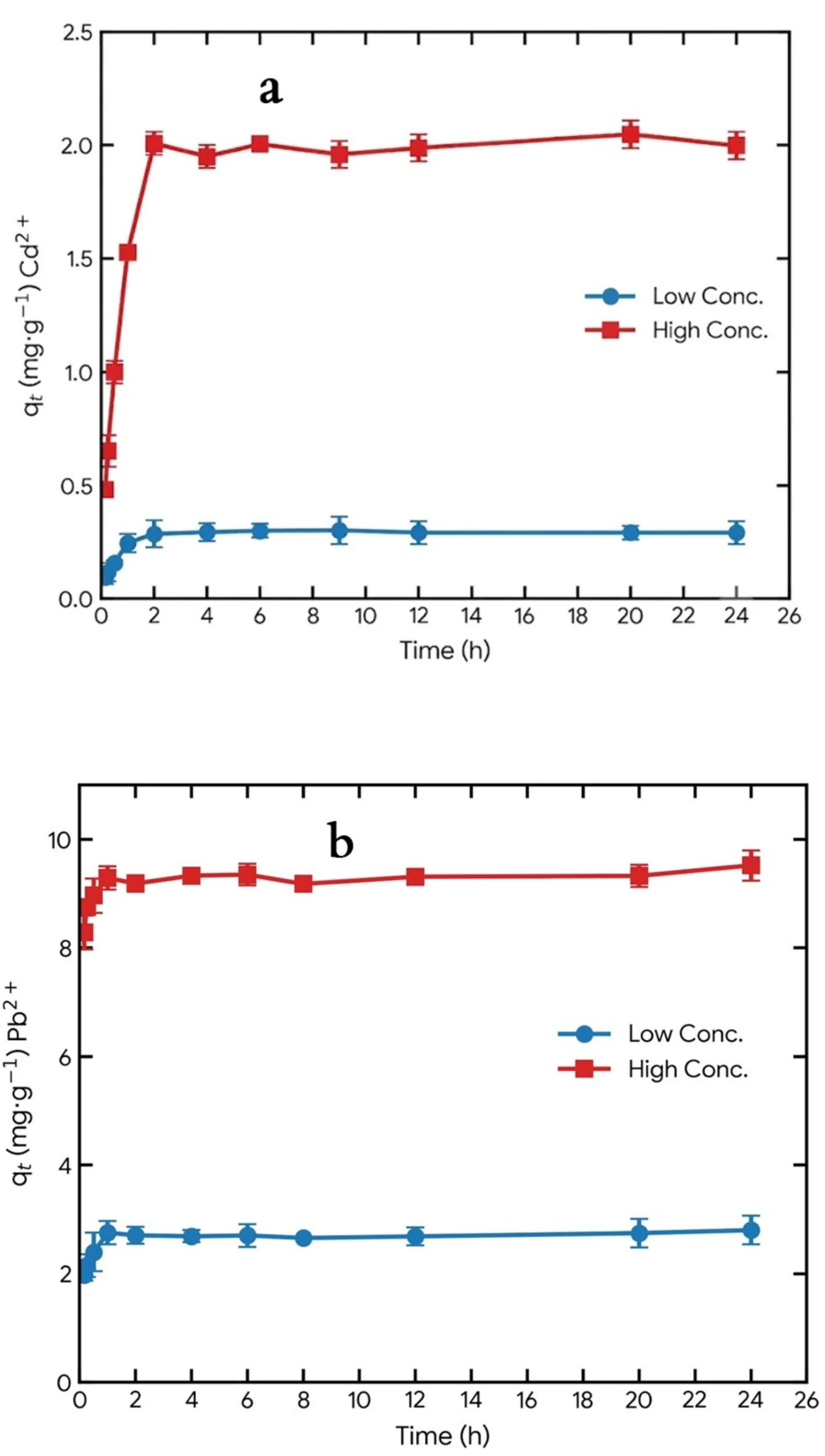
(**a**,**b**) Effect of contact time on adsorbed amount of Cd^+ 2^ and Pb^+ 2^ at two concentrations.

For cadmium and lead a kinetic study was conducted at two concentrations (low and high) for each element. For cadmium the two concentrations were (around 5 and 25 mg. L^−1^ Cd^2+^) and for lead were (around 25 and 50 mg. L^−1^ Pb^2+^). Figure 3a, b showed the adsorption of Cd^2+^ and Pb^2+^ at two concentrations. The selection of the metal concentrations in this study was based on their concentrations in selected polluted soil. The data demonstrated that the adsorption could be divided into two stages: first stage, was observed during the first 30 min, which represented the rapid adsorption phase due to fast diffusion of Cd^2+^ or Pb^2+^ from the bulk solution to the SCBB surface where adsorption sites are. The second stage corresponded to the slow adsorption phase, occurred between 30 and 120 min for Cd^2+^ and from 30 to 60 min for Pb^2+^, which represented rate-limited time-dependent process for the two concentrations. The study suggests that the first stage was attributed to the use of the accessible adsorption sites instantaneously and to the presence of several unutilized adsorption sites on the surface of SCBB which, was in agreement with Sun et al.^97^. Whereas, the migration of ions into SCBB’s pores and micro channels may be responsible for the second stage. This interpretation was consistent with Salem^62^; Shin et al.^90,91^ and Imessaoudene et al.^98^ who explained kinetic adsorption of divalent ion (Sr^+ 2^) on biochar. However, Li^99^ and Xu et al.^100^ demonstrated that the slow phase of Cd^2+^and Pb^2+^ adsorption on different types of biochar was due to a decrease in the number of possible adsorption sites gradually as the reaction time grew. Likewise, the data in Fig. 3a, b showed that the equilibrium was achieved after 2 and 1 h for Cd^2+^ and Pb^2+^ respectively, when the high quantity of adsorbed ions by SCBB was recorded and did not change over the reaction time. Physicochemical properties, FTIR, and SEM of SCBB may explain these findings, where the presence of different functional groups like –C–H and C ≡ C triple bonds in addition to, function groups, that contain oxygen–C = O, –OH, played an important role in binding these elements ^101^. C = O, OH and C–O could be a part of the adsorption of Cd^2+^ and Pb^2+^ on biochar as mentioned by Li et al.^102^ and Yu et al.^103^ when they studied the adsorption of these ions on modified lotus stem and sulfonated biochar, respectively. Thus, C = O and –OH groups could establish stable surface complexes with heavy metal ions and could adsorb heavy metals by ion exchange and electrostatic attraction, as demonstrated by Huang et al.^104^. Additionally, high specific surface area and elevated carbonization, polarity, and strong aromaticity of SCBB Table 1 may play a vital role in adsorption of these heavy metals. However, Huang et al.^104^ stated that the influence of functional groups containing oxygen on Cd^2+^ and Pb^2+^ adsorption by biochar exceeds that of specific surface area.

### Impact of initial concentration on the effectiveness removal of cadmium and lead ions by SCBB

**Fig. 4:**
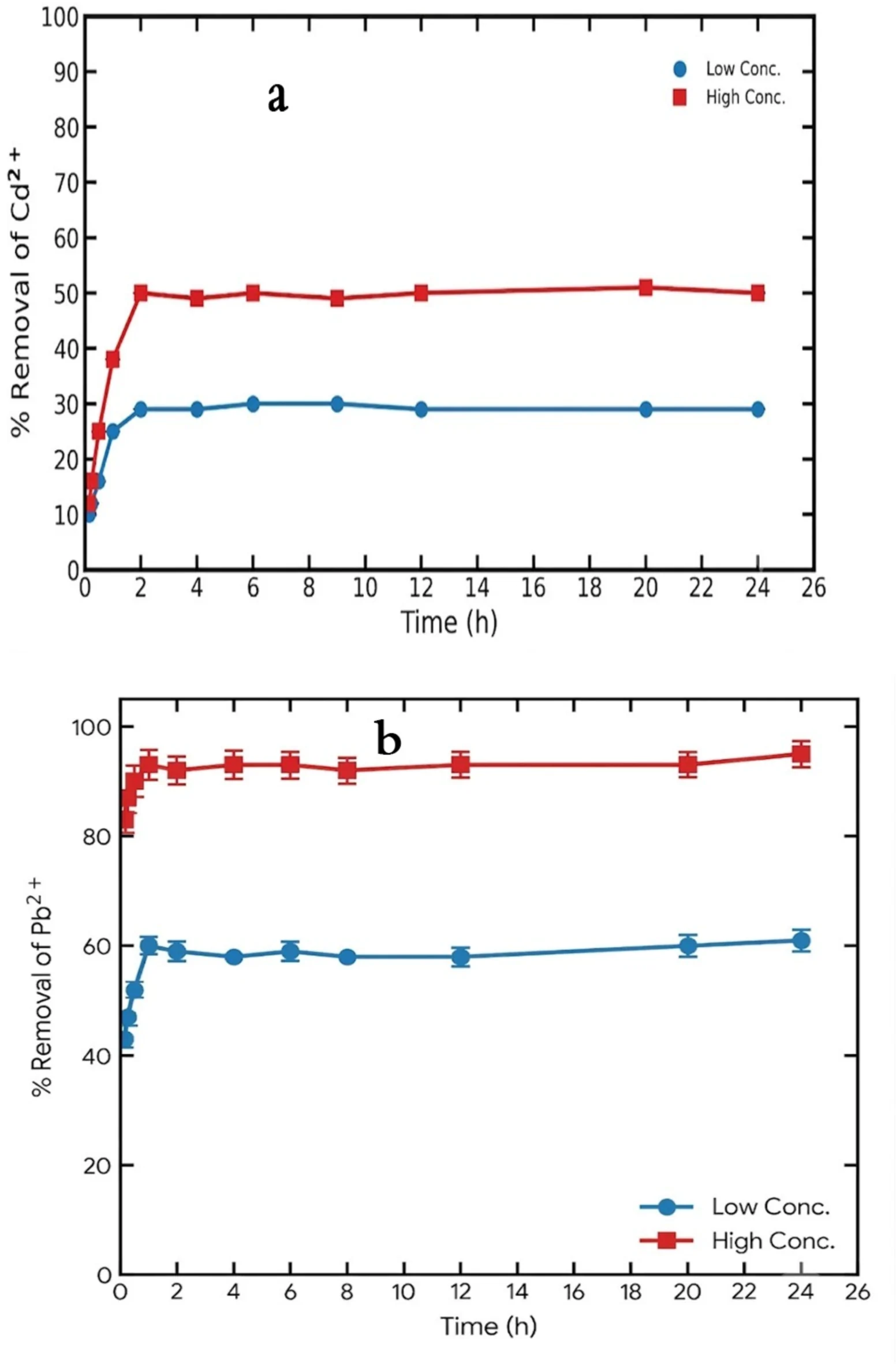
(**a**,**b**) Effect of initial concentration on the removal of cadmium and lead ions.

For Cd^2+^ the maximum percentages of removal at equilibrium were around 50 and 29% for high and low concentrations, respectively; however, for Pb^2+^, the percentages of removal were around 93% and 60% for high and low concentrations, respectively (Fig. 4a,b). The data demonstrated that SCBB acts as a good biosorbent material for Pb^2+^, more than for Cd^2+^. This was evident where SCBB had greater affinity to remove Pb^2+^ from aqueous solutions starting from the first time of contact. Additionally, at the same concentration of two elements, 25 mg. L^−1^the removal efficiency of Pb^2+^ by SCBB was greater than that for Cd^2+^ which, may be due to the ability of Pb^2+^ to form inner sphere surface complexation or other sorption reactions presumably because it has a greater ionic radius, electronegativity, atomic weight, and low hydrated ionic radius than Cd^2+^. These results were in agreement with Zhang et al.^105^; Hu et al.^106^; Huang et al.^104^ and Yang et al.^107^ who stated the high affinity of Pb^2+^ to different types of biochar compared to Cd^2+^. Likewise, the results clarified that, the percentage of removal was greater at high concentrations than at low ones for two elements at the same contact time. This phenomenon could be attributed to the fact that an upsurge in the concentration of metal ions makes it easier for them to reach the available adsorption sites on biochar. In other words, at high concentrations of metals, the concentration gradient between the bulk solution and the SCBB surface is the fundamental driving force behind high metal concentrations in adsorption. This gradient is a physical force that encourages metal ions to flow from a greater concentration (in the solution) to a lower concentration (on the surface of the adsorbent) which, increases the rate of metal ion diffusion within the adsorbent material^108^. These results were confirmed by Syeda et al.^109^; Salem^62^; Rangabhashiyam et al.^110^ who studied the adsorption of different elements on various types of biosorbents.

### Adsorption kinetics simulation

**Fig. 5:**
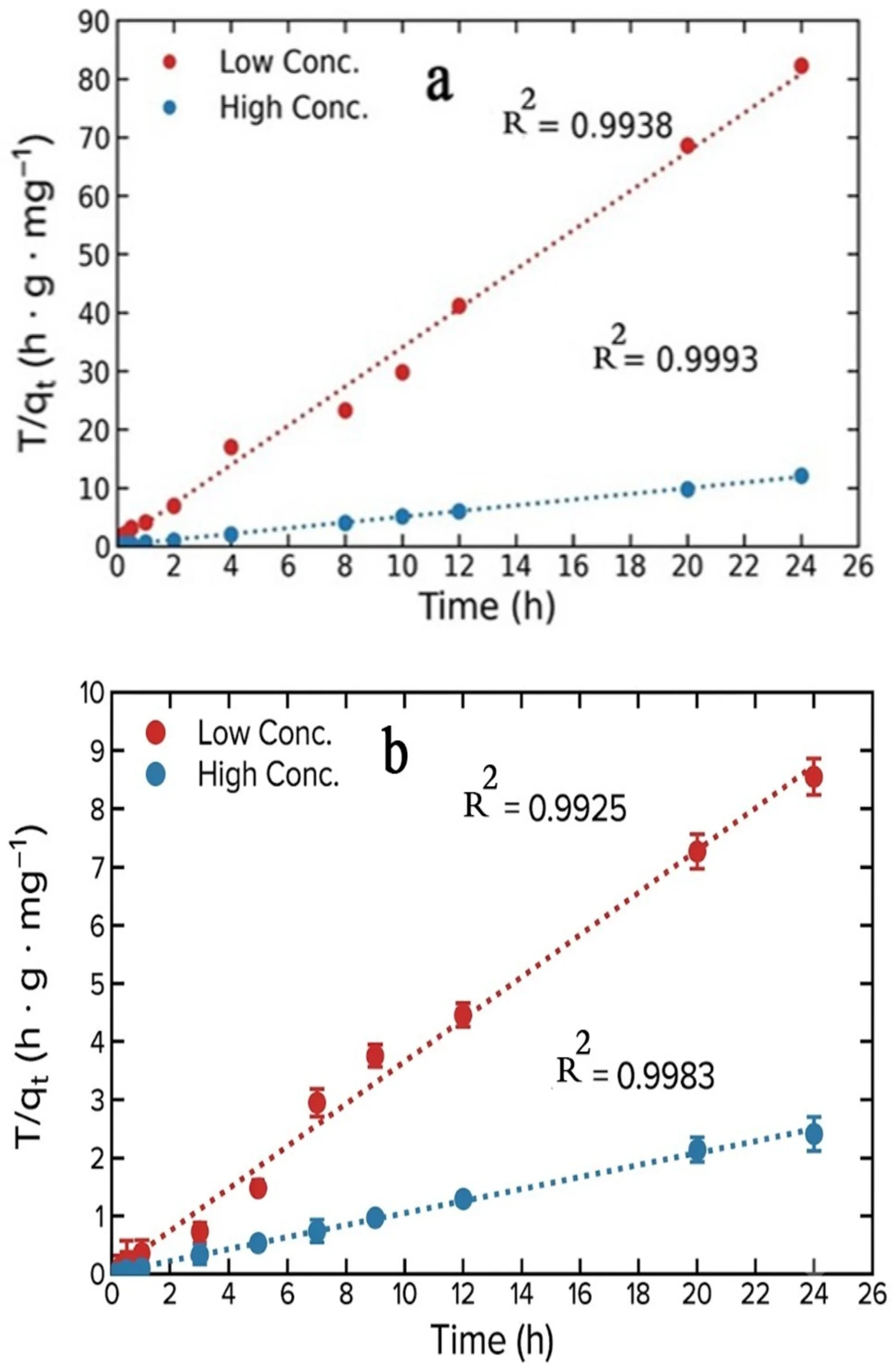
(**a**,**b**) Pseudo- second - order kinetics plot of Cadmium (**a**) and Lead (**b**) ions adsorption onto SCB biochar at two concentrations.

The study employed a Pseudo-first-order model, a Pseudo-second-order model, and an Elovich model in linear form to simulate the adsorption kinetics. Table 2 showed R^2^ and calculated parameters for these three models. According to R^2^ values and calculated parameters for these models, the Pseudo-first-order model could not simulate kinetic data where low correlation coefficients for low and high concentrations were observed for Cd^2+^ (R^2^ = 0.376 and R^2^ =0.335) respectively, while for Pb^2+^ they were (R^2^ = 0.311 and R^2^ = 0.331). The Pseudo-second-order model was more fitted to the kinetic adsorption data of Cd^2+^ and Pb^2+^ onto SCBB than other models, where the correlation coefficients were (R^2^ = 0.99 and R^2^ = 0.99) for Cd^2+^ and for Pb^2+^ were (R^2^ = 0.99 and R^2^ = 0.99) at low and high concentrations respectively. Additionally, the theoretical q_e_ calculated using the pseudo-second-order model was similar to the actual q_e_ measured for the two elements. The pseudo-second-order kinetic model describes chemisorption as a process of sharing electrons between the adsorbent and adsorbate, resulting in ion exchange and covalent forces^111,112^. Thus, chemisorption processes e.g., co-precipitation, and complexation further to other chemical reactions, Bal and Özer^113^ may govern the retention of cadmium and lead ions by SCBB, and it might be the rate-limiting stage in the adsorption process. These results were in agreement with Li^99^ who studied adsorption of Cd^2+^ and Pb^2+^onto Mg-modified straw biochar, and Liu and Fan^114^ who studied adsorption of Cd^2+^ onto wheat straw biochar. Furthermore, the rate constant of the pseudo-second-order model for Pb^2+^ was less than that of Cd^2+^ Table 2 which refers to that SCBB has a higher affinity to adsorb Pb^2+^ than Cd^2+^ and attains adsorption equilibrium faster than Cd^2+^ Fig. 3a, b^115^. That might be due to that Pb^2+^ has a smaller hydration radius in aqueous solution than that of Cd^2+^. Hydration energy increases as the hydrated ionic radius decreases. Thus, if the hydrated ionic radius decreases, the removal efficiency of metal ions increases^116^. The Elovich model describes how heterogeneous diffusion is regulated by the diffusion factor, reaction rate, and chemisorption^117^. The correlation coefficient calculated by using the Elovich model ranged between 0.7 and 0.8 for the adsorption of two elements onto SCBB. Therefore, the correlation coefficient value is higher than that of the pseudo first order model, which illustrates the importance of chemical interactions throughout the current study. On the other hand, α values for Pb at high and low concentrations were very high compared to that of Cd^2+^, which may reveal efficient interaction between SCBB and Pb^2+^. Based on what was mentioned by Sparks^101^, the reaction rate could be increased by a decrease in β and/or an increase in α, where the constants α and β have been used to estimate reaction rates (Fig. 5).

**Table 2:** Kinetic models parameters for Cd^2+^ and Pb^2+^ adsorption onto SCBB at high and Low concentration.

Conc.		Elovich			Pseudo-first order			Pseudo-second order			q_e_,exp (mg/g)
		α	Β	*R* ^2^	q_e_,_cal_(mg/g)	k_1_	*R* ^2^	q_e, cal_ (mg/g)	k_2_	*R* ^2^	
Cd^2+^	High	0.25	3.07	0.850	4.35	0.0362	0.335	2.05	1.41	0.99	2.00
	Low	0.099	24.51	0.809	2.42	0.055	0.376	0.299	20.50	0.99	0.29
Pb^2+^	High	2.15 × 10^20^	5.85	0.727	0.118	0.0253	0.311	9.67	0.639	0.99	9.29
	Low	2.55 × 10^5^	7.60	0.708	0.892	0.0166	0.331	2.71	7.22	0.99	2.76

### Adsorption isotherm study

**Fig. 6:**
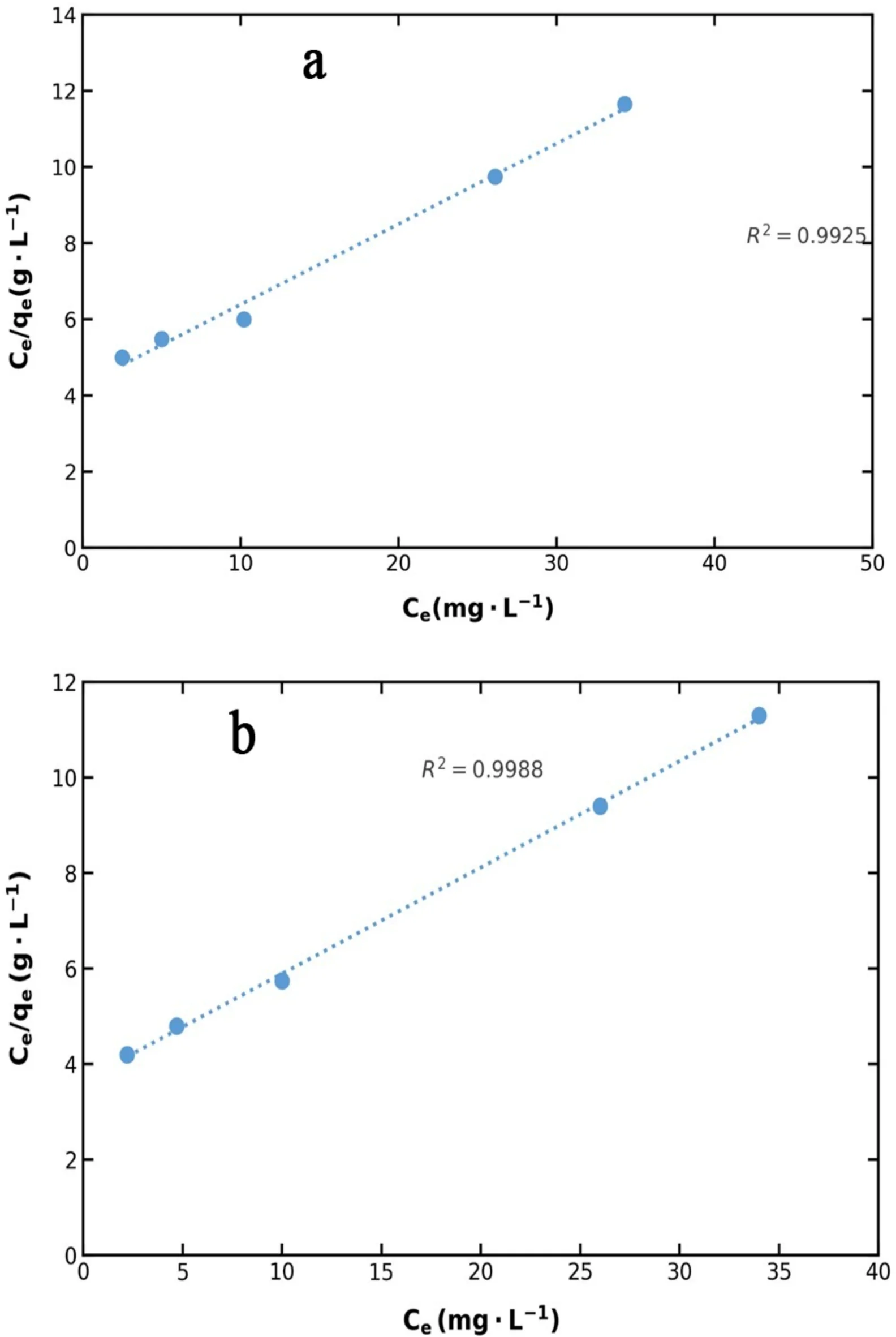
(**a**,**b**) Langmuir model in linear form for adsorption of Cd^2+^ (**a**) and Pb^2+^ (**b**) on SCBB.

 initial metal ion concentrations, ranged between 5 and 50 mg. L^−1^ for Cd^2+^ and Pb^2+^ were used to investigate their effect on adsorption at equilibration time obtained from a previous kinetic experiment. Langmuir and Freundlich isotherm models were employed to analyze the adsorption on SCBB . Figure 6a, b showed the Langmuir model for adsorption of cadmium and lead ions on SCBB.

According to correlation coefficients, the data was a better fit to the Langmuir model than Frendlich model Table 3 for Cd^2+^ and Pb^2+^. The Langmuir model suggests that Cd^2+^ and Pb^2+^ adsorption may occur via monolayer adsorption pattern on SCBB, which may be attributed to the homogeneity of the adsorbent surface and its large surface area Table 1. These findings were consistent with^44,87,118,119^. Additionally, the separation factor (R_L_) is usually utilized for describing the adsorption isotherm of the Langmuir model. For the two elements, R_L_ is within the 0 < R_L_<1 range, where for Cd^2+^ the values were ranged from 0.81 − 0.29 and were ranged from 0.77 − 0.25 for Pb^2+^. Thus, adsorption of these two elements onto SCBB was found to be favorable.

**Table 3:** Langmuir and Frendlich parameters for cadmium and lead adsorption isotherm.

Metal	Langmuir				Frendlich		
	q_max_, (mg g^–1^)	K_L_ (Lmg^–1^)	*R* ^2^	*R*_L_	*n*	K_F_ (Lmg^–1^)	*R* ^2^
Cd²⁺	4.76	0.049	0.99	0.81 − 0.29	1.47	0.29	0.97
Pb²⁺	4.50	0.06	0.99	0.77 − 0.25	1.57	0.35	0.98

### Soil characterization

According to the data in Table 4, the two soils under study have a sandy clay loam and sandy loam texture for S(I) and S (II), respectively. S(I) has a higher clay content than S(II). Additionally, S(I)has relatively more organic matter than S (II), which could lead to a higher cation exchange capacity in S(I) (10.68 meq/100 g soil) compared to S (II) (9.69 meq/100 g soil). S (II) has a higher content of carbonate (28.87 ± 2.16%) than S(I) (0.30 ± 0.03%). This difference is mostly explained by the composition and natural state of the Burg Al-Arab soil’s parent material. Accordingly, S (II) soil is more alkaline (pH = 8.65 ± 0.11) than S(I) soil (pH = 7.83 ± 0.03)^120^. Total and DTPA- extracted of some heavy metals are shown in Table 5. The results showed that the two soils are considered polluted with cadmium where the maximum permissible limit by WHO^121^ for soil is 0.8 mg kg^−1^ and they have a high content of Lead.

**Table 4:** Some physicochemical properties of two polluted soil S(I) and S(II). S(I) (soil of Sahl El Husseiniya region), S (II) (Burg Al Arab soil) before biochar addition. (average ± STDEV, *n* = 3)

Location	PH 1:2.5	EC 1:2.5 dS/m	OM %	CaCO_3_ %	CEC meq/100 g	Sand %	Silt %	Clay %	texture
S(I)	7.83 ± 0.03	2.45 ± 0.15	0.94 ± 0.33	0.30 ± 0.03	10.68 ± 1.78	58.45	18.39	23.16	Sandy clay loam
S (II)	8.65 ± 0.11	2.65 ± 0.06	0.88 ± 0.43	28.87 ± 2.16	9.69 ± 1.33	78.69	5.55	15.76	Sandy loam

**Table 5:** Total and DTPA - extracted of some heavy metals in SCBB and two polluted soils S(I) and S(II) before biochar addition.

Total and DTPA- extractable heavy metals	S(I)	S (II)	SCBB
Total content of heavy metals (mg/kg)			
Cd	9.52 ± 1.31	7.81 ± 0.06	nd
Pb	60.08 ± 1.2	59.23 ± 0.25	nd
Cu	55.66 ± 2.2	57.70 ± 1.2	15.11 ± 1.1
Mn	171.11 ± 1.4	120.35 ± 0.91	39.00 ± 1.0
DTPA- extractable heavy metals (mg/kg)			
Cd	4.31 ± 2.1	2.56 ± 0.06	nd
Pb	20.14 ± 1.00	20.27 ± 0.19	nd
Cu	10.58 ± 0.4	6.42 ± 0.02	0.14 ± 0.01
Mn	17.93 ± 0.31	10.21 ± 0.04	0.69 ± 0.01

S(I) (soil of Sahl El Husseiniya region), S (II) (Burg Al - Arab soil) and SCBB (sugar cane biochar bagasse) (average ± STDEV, *n* = 3).

### Planting experiment

The pot experiment was performed by the two polluted soils to examine the role of SCBB on soil properties, and on the stabilization of cadmium and lead elements in soil in addition to, examine the role of SCBB in reducing cadmium and lead content in plants. The pot experiment was conducted at four SCBB dosages (0%, 0.5%, 1%, 2%).

### Impact of SCBB on some soil properties

**Fig. 7:**
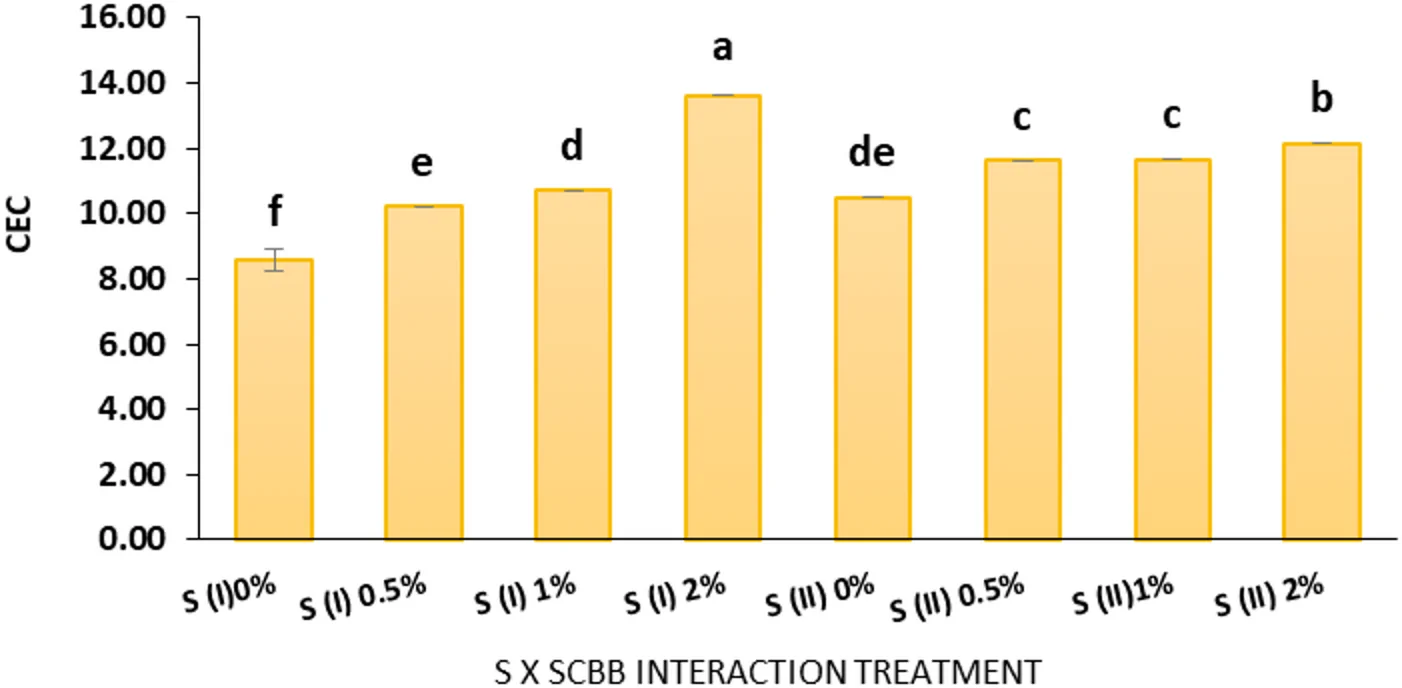
Cation Exchange Capacity (CEC, meq/100 g soil) for S(I) (soil of Sahl El Husseiniya region) and S(II) (Burg Al - Arab soil) and at four doses of SCBB (sugarcane bagasse biochar).

**Fig. 8:**
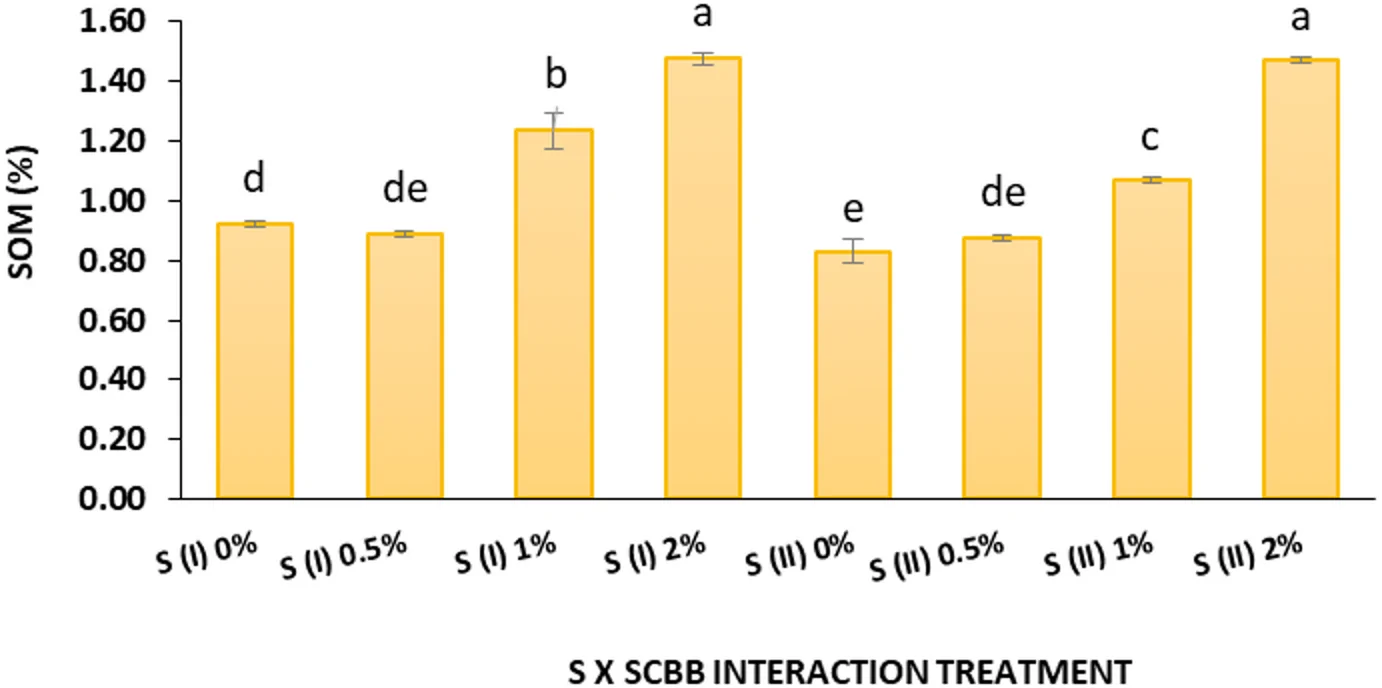
Soil organic matter content (SOM, %) for S(I) (soil of Sahl El Husseiniya region) and S(II) (Burg Al - Arab soil) and at four doses of SCBB (sugarcane bagasse biochar).

After the cultivation experiment, some soil characteristics were examined. The results demonstrated that the addition of biochar significantly (at *p* < 0.01) reduced DTPA-extracted Cd^2+^ and Pb^2+^ in S(I) and S (II), as shown in Table 6. The percentage of decrease in DTPA- extracted Cd^2+^ and Pb^2+^ was increased with increase biochar doses compared to the control. The maximum percentage of decrease corresponded to around 54% for Cd^2+^ and 51% for Pb^2+^ in S(I) that was obtained at the highest dose of SCBB (2%) where pH was ranged between 7.70 and 7.48. In S (II) the percentage of decrease was 58% and 47% for Cd^2+^ and Pb^2+^, respectively, at pH was ranges from 8.61 to 8.55. In this study, the previous kinetic results might interpret the decrease in availability of Cd^2+^ and Pb^2+^ as due to the chemisorption of them by SCBB, which has many functional groups that bind and stabilize Cd^2+^ and Pb^2+^ on its surface. Addition of SCBB to soil significantly (at *p* < 0.01) increased cation exchange capacity of soil environment, where CEC increased from 9.27 to 13.62 meq/100 g soil in S(I) and from10.50 to 12.15 meq/100 g soil in S (II) at 2% SCBB Fig. 7. Organic matter content in two polluted soils increased significantly after SCBB addition, especially at 2% dose Fig. 8; Table 6. Thus, Pb^2+^ and Cd^2^ were less mobile because soil had more metals- binding sites^122^. These findings are consistent with Bousdra et al.^123^ who found that sugarcane bagasse biochar decreased the concentration of Pb^2+^, Cd^2+^, and Cu^2+^ in the acid-extractable fraction by 40.5%, 66.6%, and 50%, respectively, which decreased the availability of metals, the study attributed this to a significant upsurge in the concentrations of residual fractions of these elements. These results were assured by Salem et al.^61^ who found an increase in residual and organic fractions of Zn²⁺, Cu²⁺, Ni²⁺ and Cr²⁺ in soil amended with SCBB. As shown in Table(6) the amount of DTPA - extracted Cd^+2^ and Pb^+2^ was less in S(II) compared to S(I) that may be due to the calcareous nature of soil in S(II) with high pH value which may enhance precipitation of cadmium and lead in carbonate and hydroxide form .Karimi et al.^124^ stated that at 2%, of corn residue biochar made at 500 °C the available amount of Cu²⁺, Zn²⁺, and Fe reduced in calcareous soil. These findings were evidenced by Shahkolaie et al.^125^ who recorded a reduction in DTPA-extractable Cd^2+^ and Pb^2+^ concentrations in calcareous soil amended with rice straw biochar and interpreted these results by increasing the oxide and organic fractions due to the high alkalinity of calcareous soil that enhances bending metals to mineral oxides and organic compounds in addition to, O-containing functional groups rich biochar. Heavy metals can precipitate with carbonate, hydroxyl, and phosphate on biochars^126^ which enhances non-electrostatic adsorption by biochar. A decline in soil pH was observed after biochar addition, which may be attributed to the presence of acidic functional groups like carboxyl and phenolic groups that raise the H^+^ ion in soil ^127^. On the other side, biochar preserves and improves the soil’s buffering capacity^128^.

**Table 6:** Some soil characteristics after SCBB treatment.

Treatment		PH	SOM	CEC	Cd- DTPA	Pb-DTPA
			%	(Meq/100gSoil) (mg/kg)		
Soil (S)						
S (I)		7.81 ± 0.01b	1.13 ± 0.07a	10.95 ± 0.55b	0.06 ± 0.01a	2.10 ± 0.07a
S (II)		8.59 ± 0.03a	1.06 ± 0.08b	11.48 ± 0.18a	0.04 ± 0.03b	0.41 ± 0.04b
Biochar (BC)						
BC_0%_		8.16 ± 0.20a	0.88 ± 0.02c	9.88 ± 0.45d	0.077 ± 0.01a	1.86 ± 0.58a
BC_0.5%_		8.14 ± 0.19a	0.88 ± 0.01c	10.92 ± 0.31c	0.057 ± 0.03b	1.24 ± 0.57b
BC_1%_		8.10 ± 0.18ab	1.15 ± 0.05b	11.18 ± 0.21b	0.042 ± 0.01c	1.01 ± 0.54c
BC_2%_		8.02 ± 0.13b	1.47 ± 0.01a	12.89 ± 0.33a	0.033 ± 0.03d	0.92 ± 0.52c
S x BC interaction						
S (I)	BC_0%_	7.70 ± 0.01e	0.92 ± 0.01d	9.27 ± 0.34f	0.099 ± 0.04a	3.16 ± 0.05a
	BC_0.5%_	7.65 ± 0.01d	0.89 ± 0.01de	10.22 ± 0.06e	0.062 ± 0.01b	2.02 ± 0.01b
	BC_1%_	7.60 ± 0.03d	1.24 ± 0.06b	10.70 ± 0.03d	0.051 ± 0.01c	1.70 ± 0.12c
	BC_2%_	7.48 ± 0.02c	1.48 ± 0.02a	13.62 ± 0.06a	0.045 ± 0.01c	1.54 ± 0.01c
S (II)	BC_0%_	8.61 ± 0.03a	0.83 ± 0.04e	10.50 ± 0.01de	0.055 ± 0.01b	0.56 ± 0.01c
	BC_0.5%_	8.62 ± 0.03a	0.88 ± 0.01de	11.62 ± 0.01c	0.053 ± 0.04b	0.46 ± 0.01 cd
	BC_1%_	8.59 ± 0.01ab	1.07 ± 0.01c	11.65 ± 0.04c	0.033 ± 0.04d	0.32 ± 0.06d
	BC_2%_	8.55 ± 0.01b	1.47 ± 0.01a	12.15 ± 0.05b	0.022 ± 0.04e	0.30 ± 0.06d
Significant test						
S		< 0.0001**	0.0005**	< 0.0001**	0.0176 *	< 0.0001**
BC		< 0.0001**	< 0.0001**	< 0.0001**	< 0.0001**	< 0.0001**
S x BC		< 0.0001**	0.0066*	< 0.0001**	0.0025*	0.0368*
CV%		0.31	3.43	1.93	12.05	5.55

Soil organic matter content (SOM) for S (I) (soil of Sahl El Husseiniya region) and S (II) (Burg Al - Arab soil) and at four doses of SCBB (sugar cane biochar bagasse). Asterisk (* and **) indicate significance at p˂0.05 and p˂0.01, respectively. According to Duncan’s multiple range test at p˂0.05 different letters denote statistically significant differences among means within each column.

### Cultivation and metal content in plant

**Fig. 9:**
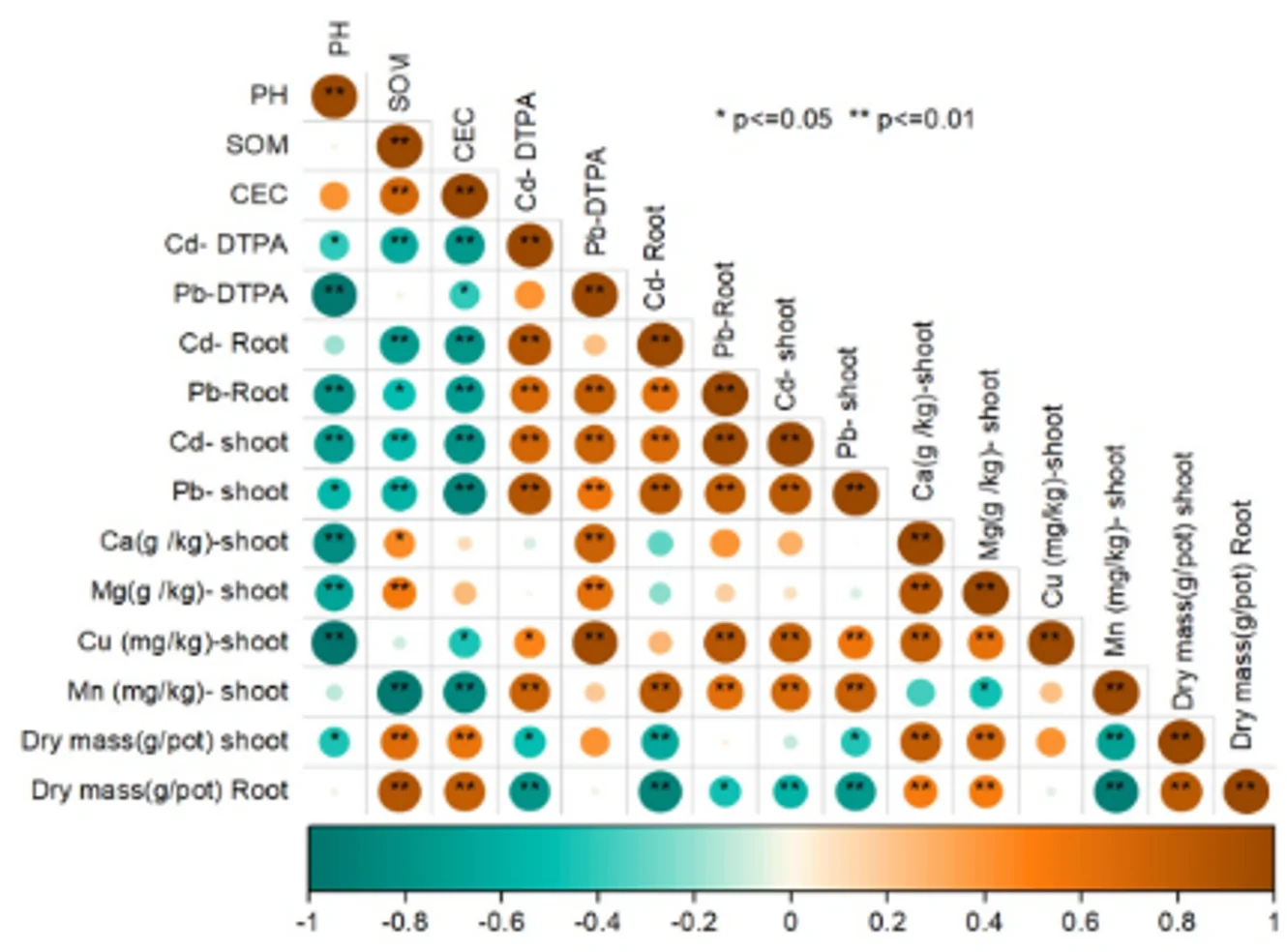
Heatmap plot correlation between the studied variables under the interaction S x BC.

The results in Table 7 demonstrated that SCBB addition significantly (at *p* < 0.01) increased shoot and root dry mass by increasing its dose. The increase in shoot dry mass was from 4.22 g/pot to 8.72 g/pot in S(I) however, in S(II) the increase in shoot was from 4.21 to 5.65 g/pot at 2% SCBB dose. For root, the increase was from 0.57 to 1.74 g/pot in S(I)and from 0.64 to 1.57 g/pot in S(II) at 2% SCBB. These results may be due to the adsorption of heavy metals by SCBB, which enhances the release, availability, and efficiency of nutrients, thereby increasing plant uptake of these nutrients and, consequently, radish dry biomass. Figure 9 shows the positive correlation (*p* < 0.01) between CEC, OM of the soil environment, and dry biomass of the shoot and root. Biochar enhanced availability of organic carbon and water retention in the soil ^129^. Salem et al.^51^ supported these results by stating that exchangeable and available macronutrients increased after the addition of SCBB and wheat straw biochar. Carter et al.^130^ and Bashir et al.^131^ observed an increase in plant biomass when soil amended with rice husk and SCBB. Moreover, Brennan et al.^132^ reported that root development was encouraged by the biochar amendment. Matheus et al.^133^ found an increase in plant biomass after application of maize cob biochar with mycorrhiza in calcareous soil. Furthermore, data in Table 8 revealed significant upsurge in Ca^2+^ and Mg^2+^ content in shoot at SCBB doses compared to control. At the highest dose of SCBB (2%), in S(I), Ca^2+^ content increased by 23%, while Mg content increased by around 14% in the shoot. Likewise, in S(II) the percentage of increase in Ca^2+^ and Mg^2+^ content in shoots was 41% and 15%, respectively. These results were consistent with Radwaniecka et al.^134^ who stated that the addition of 5% municipal sewage sludge biochar lowers the lead and cadmium concentrations in radish plants compared to the control and increases Ca^2+^ and Mg^2+^ content in plant shoots. This may be attributed to the adsorption process of HMs, including Cd^2+^ and Pb^2+^, in soil, which may interchange the numerous exchangeable metal ions, such as Ca^2+^and Mg^2+^, that are typically retained on the SCBB. This interpretation was evidenced also by Azad et al.^135^. Tables 7 and 8 showed a significant decrease in Cd^2+^ and Pb^2+^ concentrations in both aboveground and underground parts of the radish plant with SCBB treatments compared to the control. In S(I) the percentages of decline in Cd^2+^ and Pb^2+^ content in shoots were around 49% and 57% respectively, at SCBB 2%, while in root they were 55% and 59% for Cd^2+^ and Pb^2+^ respectively. In S(II) the percentage of decrease in shoots corresponded to around 54% for Cd^2+^ and 45% for Pb^2+^. In the root of the radish plant the content of Cd^2+^ and Pb^2+^ reduced by around 50% and 69% in the same order. According to the previous results, a decline in DTPA- extracted Pb^2+^ and Cd^2+^ could be reflecting this reduction in plant uptake of Cd^2+^ and Pb^2+^ in soil amended with SCBB. Where, biochar addition decreased the mobility of these ions and reduced their absorption by radish compared to control (SCBB 0%). Figure 9 the heat map correlation, showed a highly significant positive correlation (at *p* < 0.01and 0.05) between DTPA- extracted Cd^2+^ and Pb^2+^ after agriculture and their content in shoots and roots in the two soils (S(I), S(II)). These findings were consistent with results mentioned by Hao et al.^136^; Salem et al.^61^; Park et al.^137,138^; Zhang et al.^139^ who reported that biochar could reduce plant uptake of heavy metals in soil treated with different types of biochar. On the other hand, in Table 7 the data showed that Cd^2+^ tendedto concentrate more in the shoot than in the root, while Pb^2+^ concentrated more in the root. This result was consistent with Pachura et al.^140^ who found that Cd^2+^ had a high translocation factor compared to Pb^2+^ in Virginia fanpetals; the high and low mobility of cadmium and lead, respectively, from the root to the above-ground parts may lead to concentrate Cd^2+^ in the shoot and lead in the root. In S(II) the content of lead and cadmium in plant tissues may have decreased because they were bound to CaCO_3_ as mentioned previously by Bashir et al.^129^ and Lu et al.^141^ who mentioned a decrease in HMs uptake by plants in soil amended with sugarcane and rice straw biochar respectively, due to the calcium carbonate effect. According to Gong et al.^142^ the addition of biochar and lime decreased the content of Cd^2+^ and Pb^2+^ in Chinese cabbage.

**Table 7:** Cadmium and lead content in root and dry mass of shoot and root of radish plant after SCBB soil treatment.

Treatment		Cd-Root mg/kg	Pb-Root mg/kg	Dry mass-Shoot (g/pot)	Dry mass –Root (g/pot)
Soil (S)					
S (I)		0.39 ± 0.07a	3.37 ± 0.63a	6.84 ± 0.15a	1.26 ± 0.14a
S (II)		0.33 ± 0.13a	1.65 ± 0.42b	4.92 ± 0.15b	1.11 ± 0.10b
Biochar (BC)					
BC_0%_		0.53 ± 0.05a	3.45 ± 0.68a	4.22 ± 0.03d	0.61 ± 0.02d
BC_0.5%_		0.36 ± 0.03b	3.07 ± 1.11b	5.75 ± 0.38c	1.07 ± 0.01c
BC_1%_		0.29 ± 0.02c	2.27 ± 0.88c	6.36 ± 0.65b	1.41 ± 0.11b
BC_2%_		0.25 ± 0.01c	1.26 ± 0.41d	7.19 ± 0.69a	1.65 ± 0.04a
S x BC interaction					
S (I)	BC_0%_	0.61 ± 0.06a	4.21 ± 0.07a	4.22 ± 0.01f	0.57 ± 0.01 h
	BC_0.5%_	0.36 ± 0.01c	4.31 ± 0.12a	6.60 ± 0.01c	1.08 ± 0.01e
	BC_1%_	0.32 ± 0.01c	3.26 ± 0.12b	7.81 ± 0.01b	1.65 ± 0.02b
	BC_2%_	0.27 ± 0.01d	1.72 ± 0.05e	8.72 ± 0.05a	1.74 ± 0.03a
S (II)	BC_0%_	0.44 ± 0.01b	2.68 ± 0.05c	4.21 ± 0.01f	0.64 ± 0.01 g
	BC_0.5%_	0.37 ± 0.01c	1.83 ± 0.06d	4.90 ± 0.03e	1.05 ± 0.00f
	BC_1%_	0.27 ± 0.03d	1.28 ± 0.01f	4.91 ± 0.03e	1.17 ± 0.04d
	BC_2%_	0.22 ± 0.04e	0.81 ± 0.01 g	5.65 ± 0.06d	1.57 ± 0.04c
Significant test					
S		0.3358 ^ns^	< 0.0001**	< 0.0001**	< 0.0001**
BC		< 0.0001**	< 0.0001**	< 0.0001**	< 0.0001**
S x BC		0.0004**	< 0.0001**	< 0.0001**	< 0.0001**
CV%		14.04	2.55	0.88	1.25

Asterisks (* and **) indicate significance at p˂0.05 and p˂0.01, respectively; ns denotes non-significance. According to Duncan’s multiple range test at p˂0.05 different letters denote statistically significant differences among means within each column.

**Table 8:** Heavy metals and macronutrients in shoot of radish plant after SCBB soil treatment.

Treatment		Plant Shoot					
		Cd mg/kg	Pb mg/kg	Ca g/kg	Mg g/kg	Cu mg/kg	Mn mg/kg
Soil (S)							
S (I)		1.01 ± 0.08a	1.38 ± 0.49a	27.66 ± 0.67a	4.76 ± 0.11a	10.02 ± 0.40a	26.27 ± 2.15a
S (II)		0.58 ± 0.07b	1.14 ± 0.04b	17.56 ± 0.97b	3.92 ± 0.13b	2.38 ± 0.10b	26.22 ± 1.84a
Biochar (BC)							
BC_0%_		1.10 ± 0.08 a	1.94 ± 0.57a	19.31 ± 2.48d	4.13± 0.45c	6.90 ± 1.89a	33.91 ± 0.31a
BC_0.5%_		0.92 ± 0.14b	1.16 ± 0.25b	22.57 ± 2.71c	4.33 ± 0.13b	6.70 ± 1.82a	29.98 ± 0.20b
BC_1%_		0.69 ± 0.10c	1.04 ± 0.32b	24.49 ± 1.94b	4.40 ± 0.13b	6.37 ± 1.80b	24.84 ± 0.17c
BC_2%_		0.53± 0.08d	0.93 ± 0.04b	25.05 ± 1.90a	4.75 ± 0.13c	4.82 ± 1.33c	16.26 ± 0.29d
S x BC interaction							
S (I)	BC_0%_	1.28 ± 0.06a	2.30 ± 0.07a	23.86 ± 0.10c	4.55 ± 0.16b	11.11 ± 0.23 a	34.57 ± 0.20a
	BC_0.5%_	1.22 ± 0.06a	1.18 ± 0.07c	28.64 ± 0.09b	4.62 ± 0.15ab	10.76 ± 0.22ab	30.40 ± 0.15c
	BC_1%_	0.91 ± 0.06b	1.09 ± 0.12c	28.83 ± 0.09b	4.67 ± 0.23ab	10.40 ± 0.20b	24.49 ± 0.18f
	BC_2%_	0.65 ± 0.05c	0.98 ± 0.06d	29.30 ± 0.09a	5.21 ± 0.06a	7.79 ± 0.09c	15.62 ± 0.09 h
S (II)	BC_0%_	0.92 ± 0.02b	1.58 ± 0.02b	14.76 ± 0.06 g	3.72 ± 0.04e	2.68 ± 0.01d	33.26 ± 0.06b
	BC_0.5%_	0.61 ± 0.04 cd	1.14 ± 0.01c	16.51 ± 0.06f	4.04 ± 0.03c	2.63 ± 0.01d	29.56 ± 0.05d
	BC_1%_	0.48 ± 0.01d	0.99 ± 0.01d	20.16 ± 0.01e	4.13 ± 0.06 cd	2.35 ± 0.02d	25.18 ± 0.01e
	BC_2%_	0.42 ± 0.03d	0.86 ± 0.01d	20.81 ± 0.01d	4.30 ± 0.00d	1.84 ± 0.02e	16.89 ± 0.0s
Significant test							
S		< 0.0001**	< 0.0001**	< 0.0001**	< 0.0001**	< 0.0001**	0.5847 ^ns^
BC		< 0.0001**	< 0.0001**	< 0.0001**	0.0403 *	< 0.0001**	< 0.0001**
S x BC		0.0284*	< 0.0001**	< 0.0001**	< 0.0001**	< 0.0001**	< 0.0001**
CV%		9.67	4.12	0.57	3.85	3.72	0.76

Asterisks (* and **) indicate significance at p˂0.05 and p˂0.01, respectively; ns denotes non-significance. According to Duncan’s multiple range test at p˂0.05 different letters denote statistically significant differences among means within each column.

## Conclusion

The current study used sugarcane bagasse (SCB), which is a fibrous byproduct from sugar mills, to make sugarcane bagasse biochar (SCBB). The study used SCBB to investigate its role in removing Cd^2+^and Pb^2+^ from aqueous solutions. In addition, study its effect on the stabilization of these elements in two polluted soils and the decline of their bioavailability in these soils. Kinetic and adsorption isotherm experiments of Cd²⁺ and Pb²⁺ onto SCBB revealed that pseudo - second order at the two concentrations and the Langmuir model were more fitted to the results (R²=0.99) at different concentrations. In accordance with these results , chemisorption may control the adsorption behavior of Cd²⁺ and Pb²⁺ onto SCBB in aqueous solutions, and Langmuir model suggests that Cd^+2^ and Pb^+2^ adsorption may occur via monolayer adsorption pattern on SCBB. Efficiency of Pb²⁺ removal by SCBB was more than that of Cd²⁺, which were 60% and 93%, while for Cd^2+^ were 29% and 50% at low and high concentrations, respectively. DTPA- extracted Cd^2+^ and Pb^2+^ decreased in polluted soil with the addition of SCBB. 2% SCBB application rate could significantly reduce the bioavailability of Pb^2+^ and Cd^2+^ in the soil. Consequently, SCBB treatment decreased the content of Pb^2+^ and Cd^2+^ in the shoot and root of the radish plant (*Raphanus* *Sativus*). Moreover, an increase shoot and root dry mass after SCBB treatment. The study revealed an upsurge in CEC and OM in the two polluted soils. This study demonstrated that SCBB had significant potential in remediating Pb^2+^ and Cd^2+^ pollution from aqueous solutions and stabilized them in polluted soil due to its structure, physical and chemical properties. High porosity, high specific surface area, O-containing functional group-rich SCBB, and mineral composition of SCBB all contributed to biochar’s ability to retain Pb^2+^ and Cd^2+^ metals. For future soil remediation studies, long-term field experiments are recommended to enable the widespread application of SCBB at different pyrolysis temperatures to remediate Pb^2+^ and Cd^2+^ soils to avoid environmental repercussions of pollution.

## Data Availability

All data produced during this study are included in this published article.
